# Meat Freshness Monitoring in the Modern Supply Chain: Analytical Advances, Smart Packaging, and Digital Integration

**DOI:** 10.1002/fsn3.72450

**Published:** 2026-10-03

**Authors:** Md Abu Rayhan, Md. Hassan Bin Nabi, Tamanna Rahman, Md. Suhel Mia, Wahidu Zzaman

**Affiliations:** ^1^ Department of Food Engineering and Tea Technology Shahjalal University of Science and Technology Sylhet Bangladesh

**Keywords:** freshness indicators, meat spoilage detection, predictive food safety, shelf‐life monitoring, smart sensing technologies

## Abstract

Monitoring meat freshness across the modern supply chain remains challenging because meat quality changes dynamically with fluctuating cold‐chain, packaging, and logistics conditions. Conventional assessment methods, sensory inspection, physicochemical indices, and microbiological testing remain essential reference tools but are destructive, retrospective, and poorly suited to real‐time decision‐making. Growing research attention has therefore focused on sensor‐based systems, intelligent packaging, spectroscopic and imaging techniques, and digitally enabled monitoring platforms. However, many technologies that perform well in the laboratory do not scale into robust solutions under realistic supply‐chain variability. This review takes a translation‐oriented approach, reframing meat spoilage pathways in terms of their analytically detectable signals rather than their mechanistic biochemistry. Conventional freshness indices are critically assessed to explain their structural inability to operate under real logistics dynamics. Emerging technologies are evaluated not on analytical novelty alone, but on their capacity to integrate multiple spoilage signals, function under variable conditions, and support actionable freshness management, with particular attention to sensor arrays, data‐fusion schemes, IoT connectivity, machine‐learning‐based prediction, and smartphone‐assisted interpretation. This review provides a critical, criteria‐based synthesis to guide the development of robust, scalable, and decision‐oriented freshness monitoring systems capable of reducing waste, improving food safety, and strengthening supply‐chain resilience.

## Graphical abstract

Meat freshness monitoring is illustrated as an integrated system that links spoilage mechanisms to advanced detection and digital technologies. Microbial activity, enzymatic degradation, and chemical oxidation generate measurable changes in meat quality. Sensor‐based systems, spectroscopic and imaging techniques, and smart packaging solutions assess these changes. The framework highlights the progression from conventional assessment to real‐time, non‐destructive monitoring through digital integration, including data analytics, machine learning, and IoT‐enabled systems across the food supply chain.

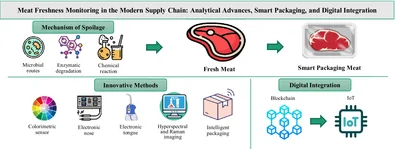

## 1 Introduction

Maintaining meat freshness is a persistent challenge across modern food supply chains, with direct implications for food safety, economic loss, and consumer trust (Li et al. 2026; Spada et al. 2024). Fresh meat quality evolves continuously from slaughter to consumption, and these dynamics are strongly shaped by cold‐chain stability, packaging atmosphere, handling practices, and logistics duration. In practice, freshness assurance has shifted from a single endpoint test to managing time‐varying risk across distributed supply‐chain nodes, which has driven growing interest in intelligent detection equipment and traceability‐oriented monitoring strategies (Wang and Zhang 2024; Zhang and Yu 2023).

Historically, meat freshness has been evaluated using sensory inspection, physicochemical indices (e.g., pH, TVB‐N, lipid oxidation markers), and microbiological counts. These methods are still critical reference tools, but they are destructive in nature, labor‐intensive, and unsuitable for continuous or in‐line monitoring. As a result, they provide limited support for rapid, actionable decision‐making in modern supply chains that require real‐time visibility and standardized, scalable workflows (Grassi and Benedetti 2023; Zhang and Yu 2023).

In response, various novel technologies have been proposed, including sensor‐based systems (e‐nose, e‐tongue, and e‐eye), intelligent packaging indicators, spectroscopic and imaging platforms, and digitally enabled monitoring systems that integrate sensing with data analytics. Recent reviews underline that e‐sensing and intelligent detection technologies have the ability to offer faster and non‐destructive assessment and can be reinforced by data fusion and higher‐order modeling, which are now more applicable in assessing the shelf‐life of animal‐derived foods (Grassi and Benedetti 2023; Wang and Zhang 2024). At the same time, systematic evidence from the meat‐packaging literature indicates that real‐world adoption is constrained by stability, calibration, interpretation, and practical deployment constraints, despite strong laboratory performance (Khodaei and Gholami‐Ahangaran 2023).

A major gap, repeatedly noted across recent work, is the translation problem: high accuracies reported in controlled experiments often do not generalize under realistic cold‐chain variability, humidity fluctuations, heterogeneous microbial ecology, and batch‐to‐batch packaging differences. This challenge is especially visible for volatile‐sensing platforms, where sensor drift and cross‐sensitivity can complicate deployment without robust calibration and modeling strategies.

Several reviews have already summarized meat spoilage mechanisms, rapid detection methods, e‐sensing systems, intelligent packaging, and non‐destructive analytical technologies. Fletcher and Mullane (2018), for example, reviewed rapid approaches to meat‐spoilage detection, whereas Grassi and Benedetti (2023) focused on e‐sensing systems for shelf‐life evaluation. Zhang and Yu (2023) reviewed intelligent detection of quality deterioration and adulteration in fresh meat products, while Khodaei and Gholami‐Ahangaran (2023) examined intelligent packaging for meat products. More recent contributions have further addressed spectroscopy and imaging (Jiang and Wang 2025; Jo and Lee 2024), electronic nose and machine‐learning applications (Shtepliuk and Domènech‐Gil 2025), and smartphone‐based freshness labels (Ehtesabi and Afkaneh 2025). These studies provide important technology‐specific foundations; however, the literature remains fragmented across sensing modalities and frequently emphasizes analytical performance rather than the conditions required for practical deployment.

A major unresolved issue is therefore the translation gap between laboratory‐level detection and supply‐chain‐level freshness intelligence. High classification accuracy, low detection limits, or strong correlations with reference measurements do not necessarily indicate robustness under fluctuating temperature, humidity, packaging atmosphere, heterogeneous microbial ecology, sensor drift, or changing meat matrices. Similarly, machine‐learning‐based systems may show strong performance under controlled datasets but exhibit reduced generalizability when applied across meat species, packaging formats, or storage conditions (Shtepliuk and Domènech‐Gil 2025). Intelligent packaging faces related challenges involving humidity, gas diffusion, calibration, interpretability, and consumer response (Khodaei and Gholami‐Ahangaran 2023; Zhai et al. 2025).

The present review therefore adopts a critical, translational, and supply‐chain‐oriented perspective. Instead of providing another technology‐by‐technology catalogue, it connects spoilage pathways with measurable signals and evaluates conventional and emerging monitoring approaches according to their ability to function under realistic operational conditions. The review specifically considers robustness, scalability, digital compatibility, interpretability, and actionability as complementary dimensions of technological readiness. This framework also enables direct comparison of sensor‐based systems, intelligent packaging, imaging and spectroscopy, and digitally integrated approaches within a common decision‐oriented context.

Accordingly, this review aims to: (i) connect major meat‐spoilage pathways with their analytically detectable signatures; (ii) critically examine the strengths and structural limitations of conventional freshness indicators; (iii) compare emerging sensing, packaging, imaging, spectroscopic, and digital technologies using common translational criteria; (iv) identify the major barriers limiting robustness, generalizability, scalability, interoperability, regulatory acceptance, and sustainability; and (v) propose a systems‐level criteria‐based synthesis for transforming freshness measurements into predictive and actionable supply‐chain intelligence.

## 2 Review Methodology and Analytical Framework

This review was designed as a structured and critical narrative synthesis of literature concerning meat freshness monitoring, with particular emphasis on the transition from conventional quality assessment toward non‐destructive sensing, intelligent packaging, spectroscopic and imaging technologies, and digitally integrated monitoring systems. The literature was organized around the central question of how freshness‐monitoring technologies can progress from laboratory‐level analytical demonstrations to robust, scalable, and decision‐oriented applications within real meat supply chains. Accordingly, the review framework considered not only analytical performance but also signal coverage, robustness, scalability, digital compatibility, interpretability, and potential for operational decision support.

### 2.1 Literature Identification and Scope

Relevant literature was identified from the references addressing meat spoilage, conventional freshness indicators, sensor‐based detection, intelligent packaging, spectroscopy and imaging, machine learning, Internet of Things (IoT) integration, and smartphone‐assisted monitoring. The literature scope included both foundational studies and recent reviews to establish the development of the field and identify persistent limitations. Particular attention was given to recent contributions addressing e‐sensing systems (Grassi and Benedetti 2023), intelligent detection systems (Wang and Zhang 2024), intelligent packaging (Khodaei and Gholami‐Ahangaran 2023), spectroscopy and imaging (Jiang and Wang 2025; Jo and Lee 2024), electronic nose and machine‐learning integration (Shtepliuk and Domènech‐Gil 2025), and smartphone‐based freshness assessment (Ehtesabi and Afkaneh 2025).

The literature was considered relevant when it addressed at least one of the following aspects: (i) biological or physicochemical signals associated with meat spoilage; (ii) conventional freshness assessment; (iii) non‐destructive sensing and biosensing; (iv) intelligent or freshness‐indicating packaging; (v) imaging and spectroscopic technologies; (vi) machine‐learning or chemometric prediction; (vii) IoT or digital integration; or (viii) practical, regulatory, economic, or sustainability barriers affecting implementation. Studies that contributed only general food‐quality information without a clear connection to meat freshness monitoring were not emphasized.

### 2.2 Thematic Organization and Evidence Synthesis

Rather than organizing the literature solely according to individual technologies, the evidence was synthesized through a signal‐to‐decision framework. First, major spoilage pathways were linked to their measurable signatures, including volatile organic compounds (VOCs), biogenic amines, pH changes, color changes, oxidative products, and spectral characteristics. This approach reflects the premise that the practical relevance of spoilage mechanisms for monitoring lies in the measurable signals they generate rather than in mechanistic description alone (Bekhit and Holman 2021; Fletcher and Mullane 2018).

Second, conventional methods were assessed according to their analytical value and their limitations under dynamic supply‐chain conditions. Sensory evaluation, physicochemical indices, and microbiological testing were therefore treated as reference or validation methods rather than automatically as deployable real‐time monitoring systems. For example, TVB‐N remains an established spoilage‐related indicator but lacks a universal threshold across meat species and storage conditions (Bekhit and Holman 2021), while lipid oxidation measurements provide valuable chemical information but do not necessarily represent microbial spoilage or consumer rejection (Abeyrathne et al. 2021).

Third, emerging technologies were compared using a common translational framework encompassing: (i) freshness signal(s) captured; (ii) detection capability and reported analytical performance; (iii) non‐destructive or in‐package operation; (iv) robustness to temperature, humidity, packaging, and matrix variability; (v) calibration and reproducibility; (vi) scalability and cost; (vii) compatibility with IoT, machine learning, or digital platforms; and (viii) potential to produce interpretable and actionable outputs. This framework was used to distinguish technologies that demonstrate high laboratory performance from those with realistic potential for supply‐chain deployment.

### 2.3 Critical Appraisal Framework

The literature was critically examined using four overarching dimensions: robustness, scalability, digital compatibility, and actionability. Robustness refers to the ability of a monitoring system to maintain reliable performance under realistic variations in temperature, humidity, packaging atmosphere, matrix composition, and sensor condition. Scalability considers instrumentation requirements, operational complexity, cost, maintenance, and feasibility of deployment across multiple supply‐chain nodes. Digital compatibility considers whether sensing outputs can be integrated with data analytics, IoT platforms, predictive models, traceability systems, or user‐facing interfaces. Actionability refers to the ability of the technology to generate outputs that can support practical decisions, such as freshness classification, remaining shelf‐life estimation, product rerouting, discounting, or withdrawal.

This critical framework was adopted because reported analytical accuracy alone does not adequately establish technological readiness. Sensor drift, cross‐sensitivity, calibration instability, and environmental sensitivity remain important barriers for sensor‐based systems (Grassi and Benedetti 2023; Shtepliuk and Domènech‐Gil 2025). Similarly, machine‐learning models may show high predictive performance while remaining vulnerable to small datasets, overfitting, insufficient external validation, and changes in meat species, packaging systems, or storage conditions (Shtepliuk and Domènech‐Gil 2025). Thus, the present review places greater emphasis on the conditions under which reported performance is obtained and whether that performance can plausibly be translated into practical supply‐chain monitoring.

### 2.4 Review Structure and Synthesis Strategy

The resulting evidence was synthesized sequentially from spoilage pathways → detectable signals → conventional reference methods → emerging sensing technologies → digital integration → implementation barriers → future decision‐oriented systems. This structure allows the review to connect the biological basis of freshness deterioration with the analytical technologies used to detect it and, subsequently, with the digital and operational systems required to convert measurements into decisions.

The synthesis therefore does not rank technologies solely according to sensitivity, detection limits, classification accuracy, or coefficient of determination. Instead, technologies are evaluated according to the extent to which they can capture complementary spoilage signals, tolerate realistic supply‐chain variability, provide reproducible outputs, integrate with digital infrastructures, and support actionable freshness management. This approach is consistent with evidence that multi‐parameter sensing, sensor arrays, and data‐fusion strategies may be better aligned with the multifactorial and dynamic nature of meat spoilage than isolated measurements (Grassi and Benedetti 2023; Wang and Zhang 2024).

### 2.5 Distinction From Previous Reviews

Several previous reviews have provided valuable foundations for this field, but they have generally emphasized particular technological domains. Fletcher and Mullane (2018) reviewed advances in rapid meat‐spoilage detection, while Grassi and Benedetti (2023) focused on e‐sensing systems for shelf‐life evaluation. Zhang and Yu (2023) addressed intelligent detection of quality deterioration and adulteration in fresh meat products, and Khodaei and Gholami‐Ahangaran (2023) systematically reviewed intelligent packaging for meat products. More recent reviews have separately examined spectroscopy and imaging (Jiang and Wang 2025; Jo and Lee 2024), electronic nose and machine‐learning approaches (Shtepliuk and Domènech‐Gil 2025), and smartphone‐based freshness‐label technologies (Ehtesabi and Afkaneh 2025).

The present review differs by integrating these technological domains into a single supply‐chain‐oriented framework. Rather than treating sensors, packaging, spectroscopy, imaging, machine learning, smartphones, and IoT as independent technology categories, the review examines how these components can be connected across the sequence of sensing → data integration → interpretation → prediction → decision‐making. Particular emphasis is placed on the translational gap between laboratory performance and field deployment, including environmental robustness, calibration stability, generalizability, cost, interoperability, regulatory considerations, and sustainability.

Accordingly, the principal contribution of this review is not the identification of another individual freshness indicator or sensing platform. Its distinctive contribution is the critical reframing of meat freshness monitoring as a systems‐level challenge, in which analytical performance represents only one component of technological readiness. This perspective extends existing technology‐focused reviews by asking not only what can be detected, but also how reliably it can be detected under realistic conditions, how the resulting information can be integrated, and whether it can support an operational decision.

## 3 Spoilage Pathways and Their Detectable Signatures

Meat spoilage is an interactive and multifactorial process that is caused by the interplay between microbial metabolic, oxidative reactions, and endogenous enzymatic activity throughout the storage and distribution stages. Although the biochemical mechanisms underlying spoilage are well established, their relevance to modern freshness monitoring lies primarily in the measurable physicochemical and biochemical signals they generate rather than in mechanistic detail (Bekhit and Holman 2021; Zhang and Yu 2023). These indicators, including volatile organic compounds (VOCs), biogenic amines, pH changes, color change, and spectral changes, are the analytical foundations of the traditional and newly developed freshness evaluation technologies (Table 1).

**TABLE 1:** Common defects in meat products.

Defect	Meat product	Bacteria involved	Storage conditions	Spoilage effect/quality impact	References
Slime	Meats	*Pseudomonas*, *Lactobacillus*, *Enterococcus*, *Weissella*, *Brochothrix*	Aerobic, chilled	Slime forms when *Pseudomonas* reaches 10^7^–10^8^ CFU/g	(Nychas et al. 2008)
H_2_O_2_ greening	Meats	*Enterococcus*, *Weissella*, *Brochothrix*, *Weisella*, *Leuconostoc*, *Lactobacillus*	Chilled	Discoloration, off‐odors	(Borch et al. 1996; Ali and Alsayeqh 2022; Nychas et al. 2008)
H_2_S greening	Vacuum packaged meats	*Shewanella* spp., *Clostridium* spp.	Vacuum/anaerobic	Sulfide odor, greening	(Nychas et al. 2008; Pellissery et al. 2020)
H_2_S production	Cured meats	*Vibrio*, *Enterobacteriaceae*	Cured, vacuum	Sulfide odor	(Nychas and Skandamis 2006)
Sulfide odor	Vacuum packaged meats	*Clostridium*, *Hafnia*	Vacuum/anaerobic	Rotten egg smell	(Pellissery et al. 2020)
Cabbage odor	Bacon	*Providencia*	Cured	Unpleasant vegetable‐like odor	(Nychas and Skandamis 2006)
Putrefaction	Ham	*Enterobacteriaceae*, *Proteus*	Cured	Strong off‐odors, gas, discoloration	(Pellissery et al. 2020)
Bone taint	Whole meats	*Clostridium*, *Enterococcus*	Bone‐in, chilled	Deep tissue spoilage	(Nychas and Skandamis 2006)
Souring	Ham	Lactic acid bacteria, *Enterococcus*, *Micrococcus*, *Bacillus*, *Clostridium*	Vacuum/anaerobic	Decreased pH, sour taste	(Nychas et al. 2008; Ali and Alsayeqh 2022)
Off‐flavors/odors	Beef, Pork	*Brochothrix thermosphacta* , *Carnobacterium*, *Leuconostoc*, *Weissella*, *Shewanella*, *Pseudomonas*	Chilled, modified atmosphere	Main spoilage in refrigerated meats	(Zhu et al. 2022; Ali and Alsayeqh 2022)
Gas production	Beef, Pork	*Leuconostoc*, *Weissella*, *Carnobacterium*	Vacuum/modified atmosphere	Pack swelling	(Zhu et al. 2022; Borch et al. 1996)
Discoloration	Beef, Pork	*Pseudomonas*, *Shewanella*, *Leuconostoc*	Aerobic, chilled	Green, brown, or iridescent color	(Pellissery et al. 2020)
Pathogen contamination	All meats	*Salmonella*, *E. coli* , *Staphylococcus aureus* , *Campylobacter*, *Listeria monocytogenes*	All	Foodborne disease risk	(Ali and Alsayeqh 2022)
Yeast/mold spoilage	Processed meats	Yeasts, molds (various genera)	Chilled, processed	Off‐odors, visible growth	(Zhu et al. 2022)
Blown pack spoilage	Meatballs, sausages	*Klebsiella pneumoniae* , *Escherichia coli*	High CO_2_ MAP, chilled	Pack swelling, off‐odors	(Zhu et al. 2022)

Abbreviations: CFU = Colony‐Forming Units, H_2_O_2_ = Hydrogen Peroxide, H_2_S = Hydrogen Sulfide, MAP = Modified Atmosphere Packaging.

As emphasized by Wang and Zhang (2024), spoilage signals evolve continuously and are strongly modulated by cold‐chain variability, including temperature fluctuations and packaging conditions. This temporal and contextual dependence explains why static, point‐in‐time measurements often fail to reflect actual freshness status under real supply‐chain conditions.

### 3.1 Microbial Activity as the Dominant Source of Spoilage Indicators

Microbial growth is widely recognized as the principal driver of meat spoilage under refrigerated storage. Spoilage‐associated microorganisms metabolize carbohydrates, amino acids, and lipids, producing ammonia, hydrogen sulfide, short‐chain fatty acids, alcohols, and biogenic amines such as putrescine and cadaverine (Fletcher and Mullane 2018; Shashank et al. 2021). These metabolites accumulate in the meat matrix and package headspace and are closely associated with off‐odors, sensory rejection, and safety risks, as illustrated in Figure 1.

**FIGURE 1:**
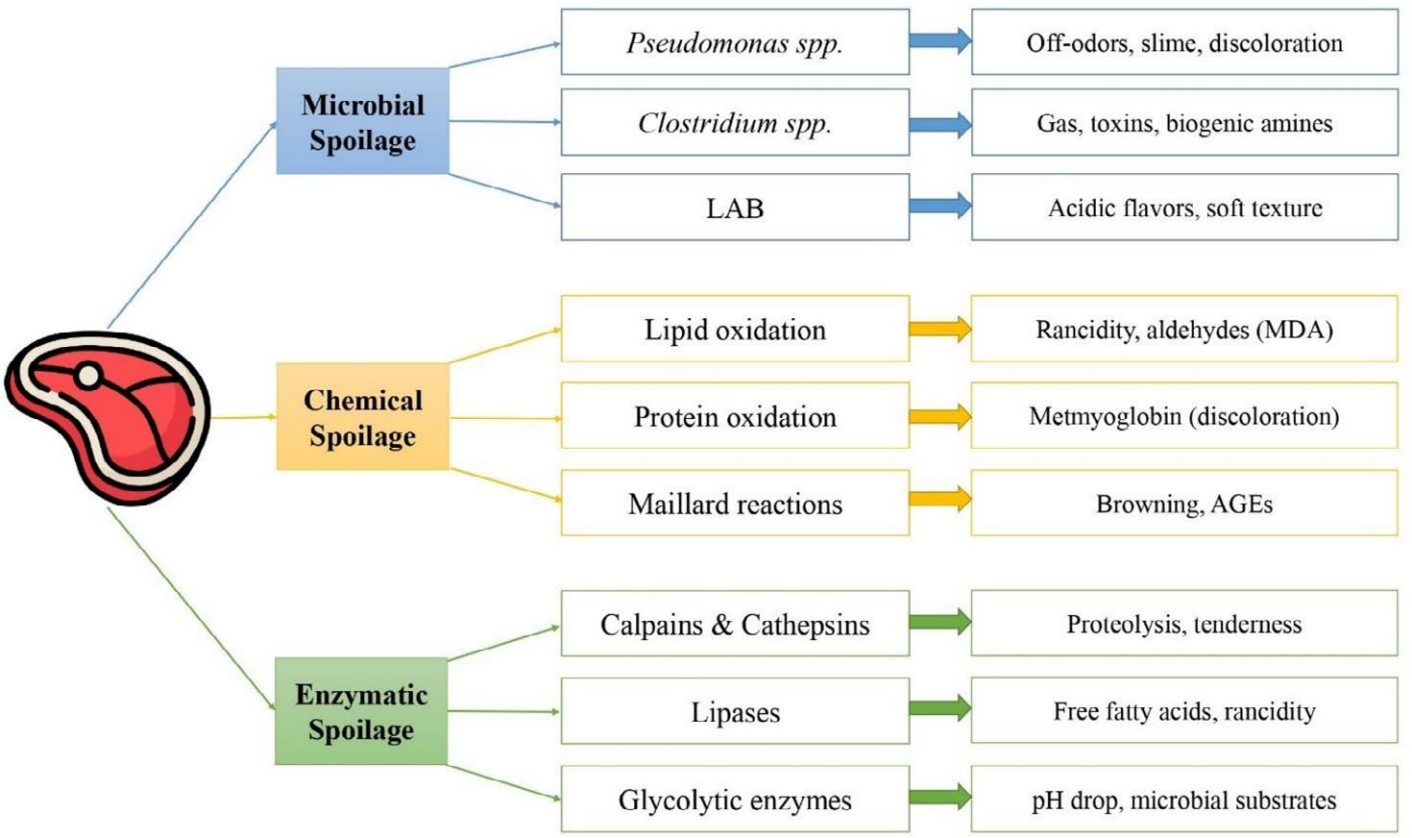
Schematic representation of microbial, chemical, and enzymatic spoilage pathways in meat and their detectable outcomes (*Source:* Created by the authors).

From a monitoring perspective, microbial spoilage is particularly relevant because it generates volatile and semi‐volatile chemical cues. Grassi and Benedetti (2023) highlighted that VOC fingerprints are among the earliest detectable spoilage signals and have therefore become key targets for electronic noses, gas sensors, and colorimetric indicators. Nonetheless, the growth of the microbes is very sensitive to the storage temperature, the atmosphere of the packaging, and the level of initial contamination, which creates high heterogeneity among products.

This inconsistency leads to the weak and inconsistent relationship that is usually realized between the total viable counts and the perceived freshness. As discussed by Bekhit and Holman (2021), absolute microbial numbers provide limited insight into spoilage intensity, reinforcing the need for sensing approaches that capture metabolic activity and signal patterns, rather than relying solely on cell enumeration.

### 3.2 Oxidative Degradation and Physicochemical Changes

Lipid, protein, and pigment oxidation are the main segments in chemical spoilage. The oxidation of lipids and proteins leads to the formation of aldehydes and ketones, which cause rancid flavors and aroma and affect water‐binding capacity, texture, and nutritional value, respectively (Abeyrathne et al. 2021). The oxidation of pigments, especially oxymyoglobin to metmyoglobin, causes visible discoloration, which has a great impact on consumer acceptance.

These oxidative processes are especially relevant for freshness monitoring because they generate optical and spectroscopic signatures. Jo and Lee (2024) established a high level of correlations between the spectral characteristics of meat discoloration and the oxidation‐related changes by hyperspectral imaging. Additional spectroscopic studies examined by Jiang and Wang (2025) also indicated that before changes in oxidation can be perceived by the senses, infrared and Raman spectroscopy are capable of detecting them at the molecular level.

Unlike microbial spoilage, oxidative degradation may progress even at relatively low microbial loads, particularly under oxygen‐permeable packaging or light exposure (Wang and Zhang 2024). Consequently, oxidation‐related signals can be precursors of visible spoilage of sensory experience and are useful early signs of non‐destructive monitoring technology.

### 3.3 Endogenous Enzymatic Activity as a Modulating Factor

Endogenous enzymes contribute to post‐mortem tenderization and structural modification during early storage but also indirectly facilitate spoilage by releasing substrates that support microbial growth and oxidative reactions. According to the summary made by Bekhit and Holman (2021), proteolysis and lipolysis are mostly modulators of spoilage kinetics, but not drivers.

Analytically, enzymatic activity is not so much a direct prospect of sensing as a generator of secondary effects of measurable properties such as pH change, water movement, drip loss, and integrity. These changes can be captured indirectly using mechanical testing, low‐field nuclear magnetic resonance, and hyperspectral imaging (Jiang and Wang 2025; Jo and Lee 2024). Detailed enzymatic pathways, therefore, add limited value to freshness monitoring unless they translate into detectable macroscopic or spectral signals.

### 3.4 Interactions Among Spoilage Pathways

As shown in Figure 2, in real meat systems, microbial, chemical, and enzymatic spoilage pathways are tightly interconnected. Enzymatic degradation increases the availability of amino acids and fatty acids, accelerating microbial metabolism and VOC production, while microbial activity alters pH and redox conditions that influence oxidation kinetics and pigment stability (Fletcher and Mullane 2018; Zhang and Yu 2023).

**FIGURE 2:**
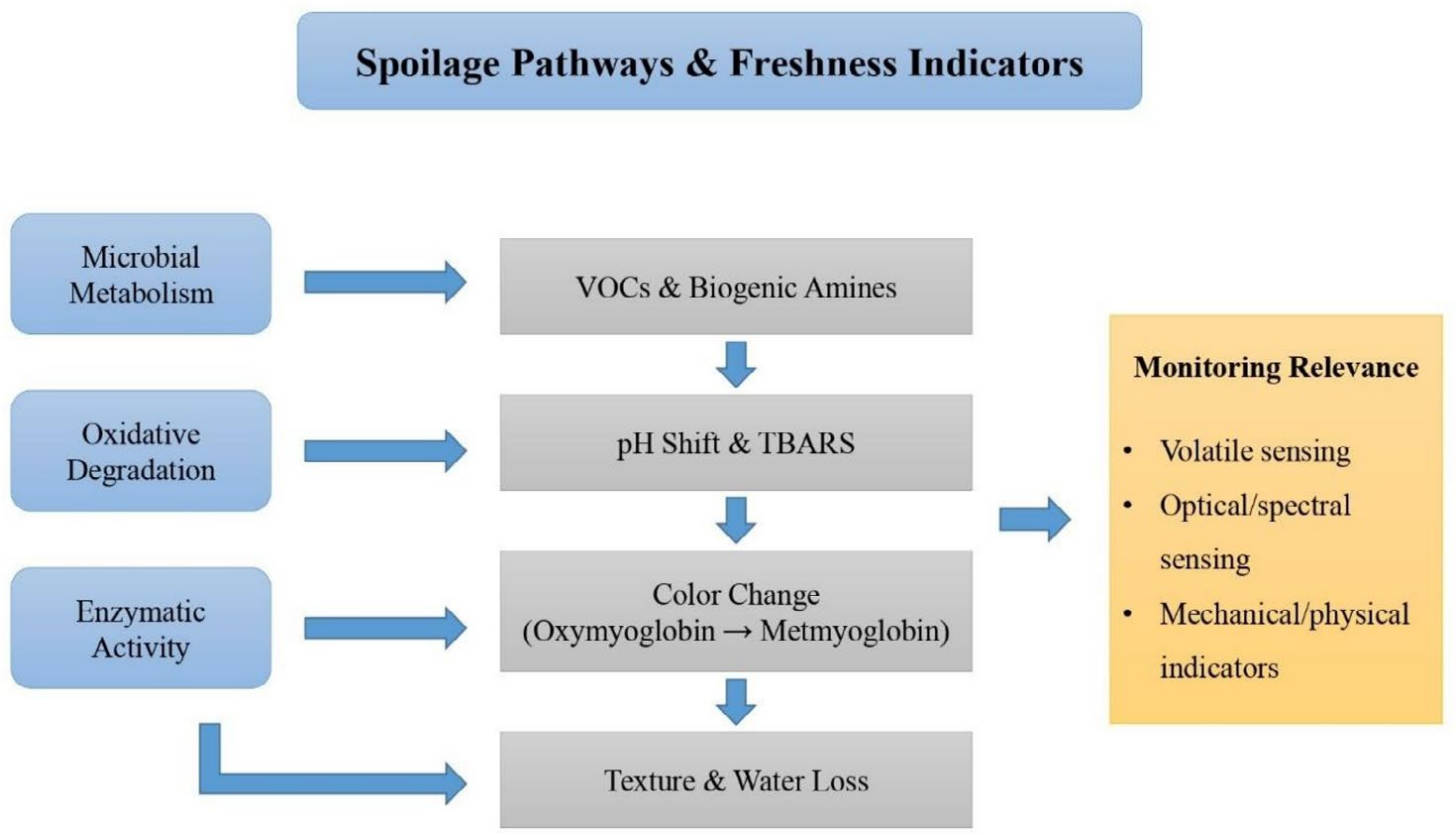
Spoilage pathways and measurable freshness signals in meat (*Source:* Created by the authors).

The consequences of these feedback mechanisms are non‐linear spoilage behavior, which differs between species of meat, packaging systems, and storage environments. Therefore, no single measure can be used dependably to measure the progress of spoilage in a variety of settings. As argued by Grassi and Benedetti (2023), effective freshness assessment increasingly relies on multi‐parameter sensing strategies, sensor arrays, and data‐fusion approaches capable of integrating complementary spoilage signals.

These pathways are also not uniform across meat types. Beef and pork spoilage under chilled, aerobic, or modified‐atmosphere conditions is typically dominated by *Pseudomonas, Shewanella, Brochothrix thermosphacta*, and lactic acid bacteria, producing the characteristic off‐flavors, discoloration, and gas production summarized in Table 1. Poultry, with a higher surface‐to‐volume ratio and different lipid composition, is comparatively more susceptible to rapid microbial proliferation and lipid oxidation under similar storage conditions. Fish and other aquatic products differ further, with spoilage often dominated by distinct microbial ecologies and more rapid biogenic amine and volatile production, which has motivated species‐specific colorimetric and fluorescent freshness indicators developed specifically for fish rather than red meat (Zhang et al. 2025). Because microbial ecology, lipid oxidation kinetics, and dominant spoilage metabolites differ across meat and seafood categories, a monitoring technology validated on one product type cannot be assumed to transfer directly to another without re‐calibration and re‐validation.

Empirically, this argument is supported by reports that combining volatile, optical, and environmental signals reduces the sensitivity of freshness classification to sensor drift, cross‐sensitivity, and matrix variability that limit single‐marker approaches, since complementary modalities can compensate for the failure or degradation of any one sensing channel (Grassi and Benedetti 2023; Shtepliuk and Domènech‐Gil 2025). Single‐marker methods such as pH or TVB‐N, by contrast, each capture only one facet of the interconnected microbial‐oxidative‐enzymatic process described above, which explains their comparatively weak and inconsistent correlation with overall sensory freshness (Bekhit and Holman 2021; Zhang and Yu 2023).

### 3.5 Implications for Freshness Monitoring Technologies

The key insight from spoilage biology is that meat freshness is defined by the convergence of multiple, time‐dependent signals, rather than by any single marker. Conventional indices such as pH, TBARS, or microbial counts each reflect only a fraction of the spoilage process and are often poorly aligned with real‐time monitoring needs (Bekhit and Holman 2021; Wang and Zhang 2024).

To this end, freshness monitoring technologies are becoming more concentrated on the acquisition of integrated signatures, such as VOC profiles, optical changes, spectroscopic fingerprints, and not quantifying individual biochemical reactions (Wang and Zhang 2024). This signal representation model offers the conceptual continuum between classical spoilage science and the intelligent, sensor‐based, and digitally integrated monitoring schemes of later paragraphs.

### 3.6 Distinguishing Spoilage Indicators From Food Safety Risk

Freshness and food safety are related but analytically distinct concepts. Spoilage indicators such as VOC profiles, biogenic amines, and pH shifts primarily reflect the metabolic activity of spoilage‐associated organisms, which govern sensory rejection but are not necessarily correlated with the presence of foodborne pathogens (Ali and Alsayeqh 2022; Pellissery et al. 2020) (Table 1). Meat contaminated with pathogens such as *Salmonella*, *Listeria monocytogenes* , *Escherichia coli* , or *Campylobacter* may still appear organoleptically acceptable and be classified as “fresh” by conventional or sensor‐based spoilage indices, as pathogen proliferation does not necessarily coincide with the activity of dominant spoilage microbiota or produce comparable volatile signatures (Ali and Alsayeqh 2022; Casaburi et al. 2015; Kang et al. 2026). Consequently, the freshness‐monitoring technologies discussed in this review should be understood as tools for quality and shelf‐life management rather than substitutes for pathogen‐specific food safety verification, which continues to require targeted microbiological or molecular testing (Bekhit and Holman 2021; Zhang and Yu 2023). This distinction should be made explicit whenever freshness indicators are proposed as decision‐support tools for supply‐chain actors, to avoid conflating consumer‐perceived freshness with microbiological safety.

## 4 Limitations of Conventional Meat Freshness Assessment Methods

Conventional approaches, such as sensory evaluation, physicochemical indices, and microbiological testing, remain foundational benchmarks in meat science and regulatory practice (Figure 3). Nevertheless, their usefulness in contemporary meat supply chains is becoming narrower due to their destructiveness, slow data accessibility, inability to respond to early spoilage, and inability to work well with real‐time decision‐making models. As emphasized by Zhang and Yu (2023), these methods were largely developed to confirm spoilage after it has occurred, rather than to manage freshness dynamically across storage, transport, and retail stages. This part critically analyzes these limitations in order to explain the reason why complementary and alternative technologies are gaining more and more ground.

**FIGURE 3:**
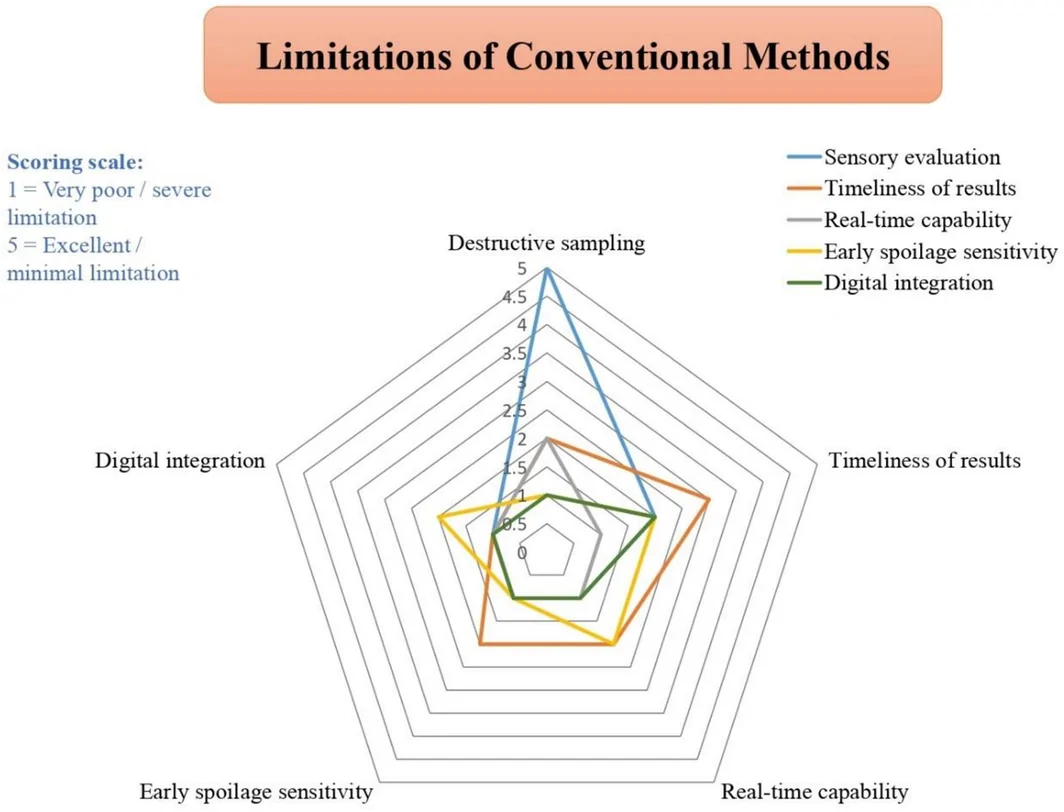
Limitations of conventional meat freshness assessment methods (*Source:* Created by the authors).

### 4.1 Sensory Evaluation: Subjectivity and Late‐Stage Detection

Appearance, odor, and texture are sensory inspection methods that are among the most frequently used freshness assessment methods because they are both quick and cost‐effective. Nevertheless, they are subjective in nature and very reliant on the training, experience, and fatigue of assessors. Fletcher and Mullane (2018) established that human perception usually signals spoilage only when the microbial loads and oxidative degradation have surpassed the acceptable levels, which is disadvantageous in the application of the method in the early phases of intervention.

Besides the delayed detection, assessor variability, and differences in environments of evaluation compromise intra‐facility reproducibility and standardization. Lighting, temperature, and personal sensitivity are also different, which makes it difficult to produce consistent results. Thus, sensory assessment is increasingly regarded as a confirmatory or rejection‐based tool rather than a proactive monitoring approach capable of enabling shelf‐life prediction or supporting cold‐chain management (Zhang and Yu 2023).

### 4.2 Physicochemical Indices: Matrix Dependence and Delayed Response

Physicochemical indices, most commonly pH, total volatile basic nitrogen (TVB‐N), and lipid oxidation markers such as TBARS, provide quantitative measures of quality deterioration and remain widely used in both research and industry. Despite their analytical robustness, these indices exhibit strong dependence on meat type, packaging atmosphere, storage temperature, and initial microbial ecology, limiting their transferability across products and operational contexts.

TVB‐N is often mentioned as an example of spoilage, but Bekhit and Holman (2021) demonstrated critically that there is no common standard. Reported acceptability limits vary substantially among species, muscle types, and packaging systems, and TVB‐N values may reflect dominant spoilage pathways rather than overall freshness status. Equally, TBARS measures the abundance of secondary lipid oxidation products and is responsive to oxygen exposure and fat content, but is not consistent with microbial spoilage or consumer rejection; instead, of integration (Abeyrathne et al. 2021).

pH measurement, while simple and standardized, often lags behind early spoilage events and may remain within acceptable ranges despite ongoing microbial and oxidative deterioration, particularly under modified‐atmosphere packaging (Fletcher and Mullane 2018). Collectively, these indices are retrospective rather than predictive, require destructive sampling and laboratory processing, and are poorly suited to continuous, in‐line, or decentralized monitoring.

Table 2 summarizes these methods comparatively: pH measurement is rapid (minutes) and minimally invasive at the point of measurement but analytically insensitive to early spoilage; TVB‐N and TBARS require destructive sampling and laboratory processing (typically hours) and lack universal acceptability thresholds; and none of the three is compatible with continuous, in‐line monitoring, in contrast to the sensor‐based and digitally integrated technologies discussed in Section 4 (Abeyrathne et al. 2021; Bekhit and Holman 2021).

**TABLE 2:** Comparative evaluation of conventional and emerging technologies for meat freshness monitoring.

Technology category	Primary freshness signal(s)	Typical numerical performance	Key advantages	Major limitations	TRL*	Digital/IoT compatibility	Relative cost/commercialization potential	References
Sensory evaluation	Odor, color, texture	Rejection typically at TVC 10^6^–10^7^ CFU g^−1^	Rapid, intuitive, no equipment	Subjective; late‐stage detection	Commercial	None	Very low cost; already commercial	(Fletcher and Mullane 2018; Hazards et al. 2020)
pH measurement	Acid–base changes	Fresh meat pH 5.5–5.8; spoiled > 6.2	Simple, standardized	Weak early‐spoilage sensitivity; matrix dependent	Commercial	Low	Low cost; commercial	(Bekhit and Holman 2021; Hazards et al. 2023; Kim et al. 2018)
TVB‐N	Volatile basic amines	Acceptability limits 15–30 mg N/100 g (species dependent)	Widely used benchmark	No universal threshold; destructive	Commercial	Low	Low–moderate cost; commercial reference method	(Bekhit and Holman 2021; Tahir et al. 2022)
TBARS	Malondialdehyde (lipid oxidation)	Acceptable 1–3 mg MDA kg^−1^	Quantitative oxidation marker	Poor correlation with microbial spoilage	Commercial	Low	Low–moderate cost; commercial reference method	(Abeyrathne et al. 2021; Domínguez et al. 2019; Yeom et al. 2025; Zhang et al. 2019)
Microbiological counts (TVC)	Viable microorganisms	Spoilage threshold 6–8 log CFU g^−1^	Regulatory relevance	24–72 h analysis; retrospective	Commercial	Low	Moderate cost (lab‐dependent); commercial, regulatory standard	(Bekhit and Holman 2021; Sanjee and Karim 2016)
Electronic nose (e‐nose)	VOC profiles	Classification accuracy 85%–98%; LOD ≈ppb–ppm VOCs	Early detection; non‐destructive	Drift; humidity sensitivity	Pilot	Moderate	Moderate instrument cost; pilot stage, early commercial units emerging	(Grassi and Benedetti 2023; Shtepliuk and Domènech‐Gil 2025)
Electronic tongue/biosensors	Amines, hypoxanthine	LOD ≈μM–mM range; accuracy > 85%–95%	High specificity	Fouling; limited lifetime	Pilot	Moderate	Moderate cost; pilot stage	(Alal et al. 2026; Aouadi et al. 2020; Zhang and Yu 2023)
Colorimetric freshness indicators	pH/amines	Visible shift at TVB‐N 10–20 mg/100 g	Simple, low cost	Visual subjectivity; humidity effects	Pilot–early commercial	Moderate	Low cost per unit; early commercial	(Lin et al. 2023; Wang et al. 2025; Zhai et al. 2025)
Intelligent packaging (TTI, gas indicators)	Time–temperature, CO_2_, NH_3_	—	Decentralized monitoring	Calibration ambiguity	Pilot	Low–moderate	Low–moderate cost; pilot, limited commercial	(Khodaei and Gholami‐Ahangaran 2023)
Hyperspectral imaging (HSI)	Spectral fingerprints	*R* ^2^ for spoilage indices 0.85–0.98	Rich spatial–chemical data	High cost; data volume	Pilot	Moderate	High instrument cost; pilot stage only	(Jo and Lee 2024; Park et al. 2026; Xing and Chen 2026)
NIR/MIR spectroscopy	Molecular vibrations	R^2^ ≈0.80–0.95 for TVB‐N/TBARS	Rapid; non‐destructive	Surface sensitivity	Pilot	Moderate	Moderate–high cost; pilot stage	(Jiang and Wang 2025; Leng et al. 2021; Qiao et al. 2017)
Raman/SERS	Molecular signatures	LOD down to μM–nM	Very high sensitivity	Fluorescence; cost	Lab–pilot	Low–moderate	High cost; lab–pilot	(Chen et al. 2024; Jiang and Wang 2025)
Nano‐enabled sensors	Enhanced VOC/chemical sensing	Sensitivity improvement compared to conventional sensors	Improved LOD	Migration and safety concerns	Lab–pilot	Moderate	Moderate–high cost (material‐dependent); lab–pilot	(Wang and Zhang 2024)
IoT‐enabled sensor networks	Multi‐signal + environment	Data latency seconds–minutes	Continuous monitoring	Infrastructure cost	Pilot	High	Moderate infrastructure cost; pilot, scalable	(Choudhary et al. 2022; Zhang and Yu 2023)
ML‐based freshness models	Pattern recognition	Prediction accuracy 85%–97%	Predictive capability	Overfitting; dataset bias	Pilot	High	Low marginal cost (software); pilot, high scalability	(Arsalane et al. 2024; Moon et al. 2020; Shtepliuk and Domènech‐Gil 2025)
Smartphone‐assisted sensing	Visual/sensor interpretation	Agreement with lab tests 80%–95%	Low cost; user engagement	Sensor‐dependent accuracy	Pilot	High	Very low cost (reuses consumer hardware); pilot, high commercialization potential	(Ehtesabi and Afkaneh 2025; Rateni et al. 2017)
Integrated multi‐modal systems	VOC + optical + environment	Shelf‐life prediction error < 10%–15%	Robust; redundant	System complexity	Pilot–early commercial	Very high	High initial cost, moderate at scale; pilot–early commercial	(Ge et al. 2026; Wang and Zhang 2024; Zhang and Yu 2023)

Abbreviations: CFU = Colony‐Forming Units, LOD = Limit of Detection, MDA = Malondialdehyde, NH_3_ = Ammonia, NIR/MIR = Near‐Infrared/Mid‐Infrared Spectroscopy, SERS = Surface‐Enhanced Raman Spectroscopy, TBARS = Thiobarbituric Acid Reactive Substances, TTI = Time–Temperature Indicator, TVB‐*N* = Total Volatile Basic Nitrogen, TVC = Total Viable Count, VOCs = Volatile Organic Compounds.

* **Experimental context:** “NR” should be used where the cited source does not provide sufficient information on meat type, sample size, storage/packaging conditions, or validation design. The reported performance values represent ranges or representative values from the cited literature and should not be interpreted as standardized head‐to‐head comparisons. **TRL classification:** In this review, TRL categories are used as a qualitative synthesis of technological maturity based on the degree of validation and practical deployment reported in the cited literature. Lab–pilot indicates controlled laboratory proof‐of‐concept with limited pilot validation; pilot indicates validation beyond basic laboratory demonstration but without established routine commercial deployment; Pilot–early commercial indicates pilot validation with evidence of initial or limited commercial applicability; and Commercial indicates established routine use in practical or industrial applications. These labels are comparative review‐level classifications and are not intended to represent formal certification of a specific numerical TRL.

### 4.3 Microbiological Testing: Accuracy Versus Timeliness

Microbiological methods, including total viable counts (TVC), psychrotrophic counts, and targeted pathogen detection, provide direct information on microbial status and remain essential for food safety verification and regulatory compliance. Nevertheless, their usefulness in the management of freshness is limited by the time of analysis and rigidity in operations. The culture‐based approach normally takes 24–72 h to produce an outcome, which does not fit into real‐time decision‐making in the dynamic logistics context (Bekhit and Holman 2021; Papkovsky and Kerry 2023).

Moreover, spoilage perception is driven more by microbial metabolic activity than by absolute cell numbers. According to Zhang and Yu (2023), the weak and inconsistent associations between TVC and sensory rejection are often reported, especially under changing cold‐chain conditions. Despite the fact that molecular and rapid methods have made the procedure fast and shortened the time of detection, they still depend on specific equipment, trained staff, and controlled laboratory conditions, which impedes scalability. This leads to microbiological testing being used as more of a verification and safety control device, and not as a continuous freshness indicator, with the ability to maintain shelf‐life forecasting or even logistics optimization.

### 4.4 Structural Limitations for Modern Supply Chains

Beyond method‐specific shortcomings, conventional freshness assessment techniques share several structural limitations. Most of them first have point‐in‐time measurements that do not represent the non‐linear, dynamic nature of spoilage in the presence of real cold‐chain variability. Second, they are destructive and hence cannot be widely deployed at various nodes along the supply chain. Third, the outputs of data are hardly digitized to correspond with computerized platforms, decision‐support programs, or predictive shelf‐life applications.

Recent syntheses by Grassi and Benedetti (2023) and Wang and Zhang (2024) emphasize that these constraints, rather than insufficient analytical rigor, are the primary drivers motivating the development of sensor‐based, non‐destructive, and digitally integrated freshness monitoring systems. Here, traditional solutions are to be considered as reference standards, whereby calibration and validation are concerned, and not as individual solutions to contemporary freshness management.

## 5 Emerging and Smart Technologies for Meat Freshness Monitoring

Expanding on the drawbacks of the traditional freshness indices as stated above, modern studies have continued to change into less destructive, signal‐based, and digitally combined monitoring methods. Nevertheless, technological innovation is not a sufficient precondition of practical relevance. As highlighted by Zhang and Yu (2023), many emerging methods replicate the same conceptual limitations as traditional indices by focusing on isolated markers or static accuracy metrics. In this part, the emerging technology is, thus, not only assessed based on the reported performance, but on how it can perform under the normal cold‐chain conditions, combine various spoilage indicators, and aid in taking action, as opposed to post hoc diagnosing.

### 5.1 Sensor‐Based Systems: Beyond Proof‐Of‐Concept Accuracy

Sensor‐based technologies, including electronic noses, electronic tongues, electrochemical biosensors, and colorimetric indicators, represent the most extensively investigated alternatives to conventional freshness assessment, presented in Figure 4. Their principal advantage lies in the ability to detect spoilage‐related metabolites such as volatile organic compounds (VOCs), biogenic amines, and hypoxanthine non‐destructively and at earlier stages than sensory or laboratory‐based methods (Grassi and Benedetti 2023; Shtepliuk and Domènech‐Gil 2025).

**FIGURE 4:**
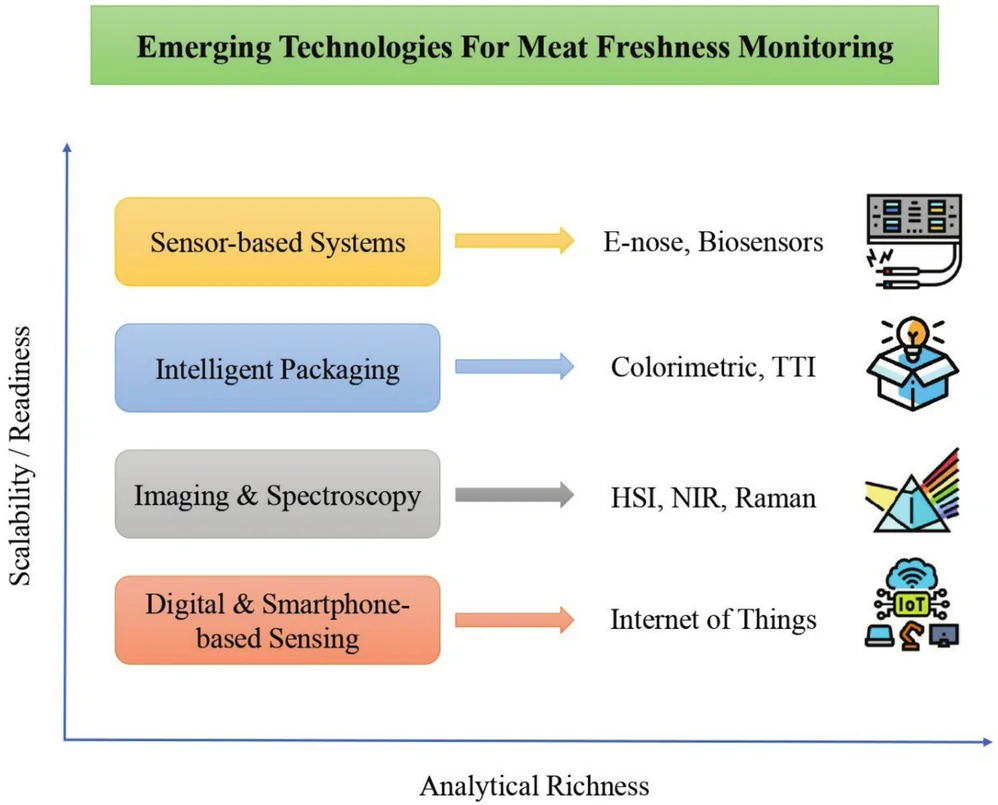
Landscape of emerging technologies for meat freshness monitoring (*Source:* Created by the authors).

Most studies of sensors are limited to laboratory conditions, even though there have been numerous reports of classification accuracies of over 90%–95%. Performance is often evaluated under constant temperature, fixed humidity, and artificially inoculated samples, conditions that poorly reflect the variability of commercial cold chains (Alal et al. 2026; Munekata et al. 2023; Wang and Zhang 2024).

These high accuracies are typically obtained using pattern‐recognition or machine‐learning classifiers (e.g., discriminant analysis or artificial neural networks) applied to sensor‐array outputs from relatively small, single‐batch sample sets, validated by internal cross‐validation rather than an independently collected external test set, and using meat matrices stored or inoculated under a single, standardized condition (Shtepliuk and Domènech‐Gil 2025). Because sample size, meat matrix, and validation strategy are rarely reported consistently across studies, the 85%–98% range summarized in Table 2 should be read as an indication of laboratory‐stage feasibility rather than a benchmark of field‐ready performance.

Recent critical analyses increasingly recognize that sensor drift, cross‐sensitivity, and calibration instability, rather than detection limits, constitute the dominant barriers to industrial adoption. As discussed by Shtepliuk and Domènech‐Gil (2025), these have inspired the move toward single‐sensor solution replacement by sensor arrays and hybrid systems, in which redundancy and data fusion can help reduce environmental noise and component degradation. Translationally, sensor‐based systems seem to be most useful when implemented as relative change sensors, which monitor the freshness development over time instead of trying to measure freshness on an absolute basis (Grassi and Benedetti 2023).

### 5.2 Intelligent and Active Packaging: Information Versus Intervention

Active and intelligent packaging serve distinct functions and should not be treated interchangeably. Active packaging is an intervention‐based strategy: it modifies the internal package environment, for example, by releasing antimicrobials or antioxidants, or scavenging oxygen to actively retard spoilage and extend product quality (Rayhan et al. 2026; Zhai et al. 2025). Intelligent packaging, by contrast, is an information‐based strategy: it embeds indicators responsive to temperature history, pH changes, or volatile metabolites to report on quality status without altering the product itself, thereby decentralizing freshness assessment across the supply chain (Zhang and Yu 2023). Although both are frequently discussed together because they are often integrated into the same packaging system, active packaging changes the product's spoilage trajectory, whereas intelligent packaging only monitors it—a distinction with practical implications for validation and regulatory pathways, since active packaging requires efficacy testing while intelligent packaging requires accuracy and reliability testing of its reporting function (Khodaei and Gholami‐Ahangaran 2023). Nevertheless, there is an inherent ambiguity in the literature related to the information‐providing and the intervention‐based packaging strategies. Although active packaging is capable of retarding spoilage by releasing antimicrobials or antioxidants, intelligent packaging only reports quality degradation. Many studies conflate these functions, overstating the practical impact of color‐change indicators without adequately addressing calibration, interpretability, or consumer response (Rayhan et al. 2026; Zhai et al. 2025).

In addition, the level of indicator performance under actual conditions is not yet well tested. According to Khodaei and Gholami‐Ahangaran (2023), humidity changes, gas diffusion laws, and light contact may also play a significant role in determining the indicator's reliability. Consequently, intelligent packaging should not be viewed as a standalone solution, but rather as a visual interface within a broader sensing and data ecosystem, particularly when combined with digital interpretation tools and predictive models.

Beyond the information‐versus‐intervention distinction, several practical factors constrain the real‐world performance of intelligent packaging indicators. Indicator response can be strongly affected by ambient humidity and headspace gas composition rather than meat freshness alone, so indicators calibrated under one packaging atmosphere may generate false positives or false negatives under another (Khodaei and Gholami‐Ahangaran 2023). Consumer interpretation adds a further layer of uncertainty: colorimetric or visual indicators rely on the end user correctly perceiving a gradual color shift, which is subject to lighting conditions and individual color perception, and may not translate into a consistent purchasing or discard decision (Nijum et al. 2025; Zhai et al. 2025; Zhang and Yu 2023). Cost is also a limiting factor for widespread adoption, since indicator materials, printing, and quality‐control steps add to per‐unit packaging cost without a guaranteed offsetting reduction in waste at the individual‐package level (Khodaei and Gholami‐Ahangaran 2023). Finally, compatibility with existing commercial meat‐packaging lines, including printing or laminating indicators onto film substrates at production‐line speeds, remains a largely unresolved engineering challenge that is rarely addressed in laboratory‐stage indicator studies.

### 5.3 Imaging and Spectroscopic Techniques: Rich Data, Limited Scalability

Imaging and spectroscopic techniques, including hyperspectral imaging (HSI), near‐ and mid‐infrared spectroscopy (NIR/MIR), fluorescence, and Raman‐based methods, provide rich molecular and spatial information related to oxidation, microbial activity, and structural changes in meat (Table 2). Under laboratory and pilot‐scale conditions, these approaches consistently demonstrate strong correlations with conventional spoilage indices and sensory quality (Jiang and Wang 2025; Jo and Lee 2024).

Although they possess this analytical ability, their practical implementation is limited by the cost of the instruments, the dimensionality of the data, and the complexity of processes. Though the current technologies in chemometrics and machine learning have increased the predictive capabilities, the key challenge in translation is the integration of systems instead of the detection ability. Spectroscopic methods will not be able to be effectively used to monitor supply chains using a distributed set without automated data pipelines, standardized calibration protocols, and simplified outputs (Wang and Zhang 2024). New attempts at integrating imaging platforms with embedded analytics, edge computing, and simpler user interfaces indicate a potential way out, but will be difficult to implement in practice until hardware complexity and cost can be reduced by significant margins (Jiang and Wang 2025).

### 5.4 Digital Integration: IoT, Machine Learning, and Smartphone‐Based Sensing

The key trend in recent years is the digitalization of freshness monitoring, that is, sensors are not judged as isolated entities but rather as the elements of a systemic network. As illustrated in Figure 5, IoT‐based systems allow obtaining real‐time environmental and freshness‐related data, and machine learning algorithms convert the complicated sensor data into predictive shelf‐life and decision‐support systems (Niam et al. 2025; Wang and Zhang 2024; Zhang and Yu 2023).

**FIGURE 5:**
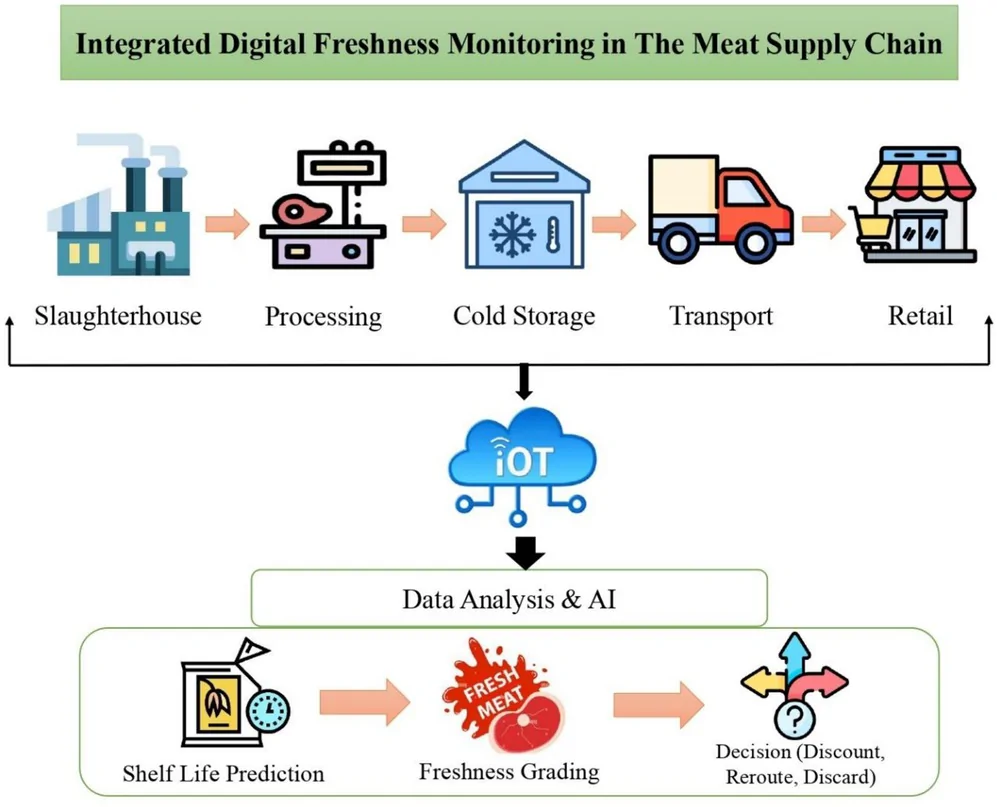
Integrated digital freshness monitoring framework across the meat supply chain (*Source:* Created by the authors).

Simultaneously, smartphone‐based freshness assessment has become one of the prospective translational avenues. Smartphones can use built‐in cameras and application‐level analytics to standardize colorimetric indicator interpretation and minimize subjectivity related to visual inspection (Zhou et al. 2026). Such systems are not used to replace analytical sensors, although they offer a low‐cost interface between intelligent packaging and end users, which has been a significant impediment to implementation. However, the existing digital systems are often affected by small datasets, overfitting, and a lack of external validation. Reported model performance often deteriorates when applied across meat species, packaging formats, or storage conditions, underscoring the need for standardized benchmarking protocols and large‐scale field trials (Shtepliuk and Domènech‐Gil 2025).

Illustrating this translational potential, smartphone‐readable label‐on‐package systems and colorimetric/fluorescent dual‐modal indicator labels have already been demonstrated as low‐cost, consumer‐ and industry‐facing formats capable of interfacing directly with existing packaging lines, moving intelligent‐packaging concepts closer to practical deployment (Ehtesabi and Afkaneh 2025; Zhang et al. 2025). Beyond predictive modeling, digital integration raises data security, interoperability, and standardization challenges that remain underexplored in the meat freshness‐monitoring literature. IoT‐connected sensing and smartphone‐based platforms generate and transmit product‐ and shipment‐level data across multiple supply‐chain actors, requiring secure data transmission and storage to prevent tampering with freshness or traceability records (Zhang et al. 2025). At the same time, the absence of standardized data formats and communication protocols across sensor vendors, packaging suppliers, and logistics platforms limits the ability to aggregate freshness data into shared decision‐support systems, reinforcing the need for the interoperable, standardized digital architectures discussed further in Section 7.3.

Reported machine‐learning approaches in this domain span a range of algorithm families, from classical classifiers such as random forest and support vector machines to artificial neural network and deep‐learning architectures applied to sensor‐array, spectral, or image data (Shtepliuk and Domènech‐Gil 2025). Model choice interacts strongly with dataset size: tree‐based and support‐vector approaches can perform competitively on the small, high‐dimensional datasets typical of e‐nose and spectroscopic studies, whereas neural‐network and deep‐learning models generally require substantially larger and more diverse training sets to avoid overfitting (Shtepliuk and Domènech‐Gil 2025). Validation strategy is equally consequential: k‐fold cross‐validation performed within a single dataset provides only an internal estimate of fit, whereas validation on an independently collected external dataset spanning different meat batches, packaging formats, or storage conditions provides a substantially more realistic estimate of field performance (Shtepliuk and Domènech‐Gil 2025; Zhang and Yu 2023). Given that many published studies rely on fewer than 100 samples, reported accuracies should be interpreted cautiously until larger, externally validated datasets and standardized benchmarking protocols become available.

### 5.5 Synthesis: What Distinguishes Promising Technologies

Across sensor‐based, packaging‐integrated, spectroscopic, and digitally enabled approaches, a consistent pattern emerges. Technologies that focus on single markers or isolated accuracy metrics rarely translate beyond laboratory demonstrations. In contrast, systems that emphasize signal integration, redundancy, and contextual interpretation are better aligned with the realities of modern meat supply chains (Grassi and Benedetti 2023; Wang and Zhang 2024).

From a systems‐level perspective, the future of meat freshness monitoring does not lie in identifying new spoilage indicators, but in integrating existing ones into robust, scalable, and interpretable systems. The analysis of emerging technologies should be viewed, then, not so much in terms of analytical novelty but in terms of their ability to aid actionable decisions with realistic variability of cold chains.

When compared in terms of cost and commercialization readiness, conventional methods remain low‐cost but are largely limited to point‐based assessments, whereas electronic nose/tongue systems and colorimetric indicators offer relatively low‐to‐moderate cost and show potential for pilot‐to‐early commercial deployment (Munekata et al. 2023). In contrast, hyperspectral and spectroscopic platforms provide high analytical resolution but are associated with substantial instrumentation costs, complex data processing, and limited suitability for routine in‐line applications (Alal et al. 2026). Overall, most emerging sensing technologies remain at the laboratory or pilot stage, highlighting a gap between analytical performance and industrial deployment (Khaled et al. 2021).

The performance values summarized in Table 2 should be interpreted within the context of their respective experimental designs rather than as directly comparable benchmarks. Reported accuracy, *R* ^2^, and LOD values are affected by meat species and muscle type, sample size, initial microbial condition, packaging atmosphere, storage temperature and duration, sample preparation, and model‐validation strategy. This limitation is particularly important for sensor‐ and machine‐learning‐based approaches, for which controlled laboratory conditions, small datasets, artificial inoculation, and insufficient external validation can lead to optimistic estimates of performance. Consequently, technological maturity in this review is considered alongside robustness, scalability, validation environment, and digital compatibility rather than numerical performance alone. This approach is consistent with the broader translational perspective of the review, which emphasizes that strong laboratory accuracy does not necessarily guarantee reliable operation under variable cold‐chain conditions.

## 6 Challenges and Limitations in Translating Freshness Monitoring Technologies

Despite rapid technological progress, the large‐scale adoption of advanced meat freshness monitoring systems remains limited. This is not mainly because of a lack of analytical performance, but rather due to technical fragility, incompatibility in operations, economic restrictions, and regulatory uncertainty. To overcome these issues, there will be a need to move the definition of optimization to the system level and to validate the system in realistic conditions of the supply chain.

### 6.1 Technical Robustness and Calibration Stability

The inability of sensing systems to withstand over time is one of the most persistent technical issues. Sensor drift, baseline instability, and cross‐sensitivity to humidity, temperature, and non‐target volatiles are widely reported across electronic noses, colorimetric indicators, and electrochemical sensors (Grassi and Benedetti 2023; Shtepliuk and Domènech‐Gil 2025). Such problems deter reproducibility and restrict the ability to compare results between studies and deployment locations.

An important weakness of the existing literature is the excessive use of short‐term performance measures, which are frequently based on in vivo laboratory settings. In real cold‐chain environments, sensors are exposed to fluctuating temperatures, mechanical vibration, and heterogeneous headspace composition, conditions rarely reproduced in validation studies (Wang and Zhang 2024). Translationally, strong recovery frequency is more applicable compared to small gains in detection limits, which are, however, less reported.

### 6.2 Data Validity, Modeling Bias, and Generalizability

Complex sensor and spectral data have been significantly enhanced in terms of interpretability with the help of machine learning and chemometric models. However, their reliability is constrained by limited datasets, overfitting, and lack of external validation. The reported accuracies are often based on relatively small datasets or on artificially inoculated meat, suggesting that they may not be applicable to meat species, packaging systems, and even storage conditions (Shtepliuk and Domènech‐Gil 2025).

In addition, there is a lack of standard benchmarking datasets that can be used to make a meaningful comparison between competing technologies. Consequently, it is still hard to tell whether some real technological advances are present or a model‐dependent inflation of performance. Without transparent reporting of training–validation splits, data diversity, and model robustness, predictive freshness systems risk remaining research artifacts rather than deployable tools (Zhang and Yu 2023).

This limitation is compounded by the fact that many reported classification or prediction accuracies are obtained under a single, fixed experimental condition: one meat type, one packaging format, one storage temperature so that a model's apparent performance reflects its fit to that specific condition rather than a generalizable freshness relationship (Shtepliuk and Domènech‐Gil 2025). When models are evaluated only on data drawn from the same distribution used for training, without a held‐out, independently collected external validation set, reported accuracy is likely to overstate real‐world performance, since it does not capture batch‐to‐batch biological variability, sensor drift between deployments, or shifts in packaging or supply‐chain conditions (Shtepliuk and Domènech‐Gil 2025). Future reporting should therefore distinguish clearly between internal cross‐validation accuracy and true external validation accuracy, and should report performance degradation across meat species, packaging systems, and temperature‐abuse scenarios rather than a single headline accuracy figure.

### 6.3 Integration Into Industrial Workflows

The other significant weakness is the inadequacy of sensor outputs in correlation to industrial decision processes. A lot of systems produce and generate complex and high‐dimensional data that can only be interpreted by experts, and this is not possible in a high‐throughput processing environment. Specifically, imaging and spectroscopic platforms are challenged with the issues of data size, processing speed, and hardware complexity, though they are highly analytical (Jo and Lee 2024).

Operationally, freshness monitoring technologies should be able to integrate with the already existing logistics, inventory management systems, and quality assurance systems. Technologies unable to provide easy and practical outputs, for example, freshness scores, shelf‐life forecasts, or binary warnings, are not likely to gain mass usage, no matter how well they are analytically developed (Wang and Zhang 2024).

The specific output format required differs by supply‐chain role. Processors and quality‐assurance teams generally benefit most from continuous, quantitative freshness scores or estimated remaining shelf‐life values that can be logged and trended over a production run, supporting root‐cause investigation when deviations occur. Retailers and distributors are better served by simplified, categorical outputs, such as traffic‐light or pass/fail warnings that support rapid stock‐rotation and markdown decisions without requiring interpretation of underlying sensor data. Logistics operators, primarily concerned with cold‐chain integrity rather than product‐level freshness per se, benefit most from threshold‐based alerts tied to temperature or time deviations that trigger rerouting or intervention before spoilage risk accumulates (Wang and Zhang 2024; Zhang and Yu 2023). Aligning output format with the decision each stakeholder actually needs to make is therefore as important as the underlying sensing accuracy.

### 6.4 Economic Feasibility and Scalability

The cost factor is also a decisive factor, especially on small‐scale and medium‐sized meat processors. The incorporation of sensor arrays, wireless modules, or smart packaging elements increases unit costs and may not be offset by immediate economic benefits. Lots of investigations provide evidence of technical feasibility with no consideration of cost–benefit trade‐offs, manufacturing, manufacturing scale, or maintenance issues (Khodaei and Gholami‐Ahangaran 2023).

Smartphone‐assisted systems and less expensive indicator technologies could be a good compromise in this scenario. These strategies will lower the deployment expenses by reusing consumer hardware and infrastructure, enhance interpretability, and improve user interaction (Zhang et al. 2025). However, their reliability still depends on upstream sensor stability and calibration consistency.

### 6.5 Regulatory and Sustainability Constraints

Regulatory approval represents a significant bottleneck for intelligent and active packaging systems, particularly those incorporating nanomaterials or migrating chemical indicators. The food‐contact policies are associated with a lot of migration tests, toxicological analysis, and labeling, which are not a priority in early‐stage studies (Khodaei and Gholami‐Ahangaran 2023).

Migration testing and toxicological assessment are particularly critical when active or intelligent packaging incorporates nanomaterials, since nanoscale components can exhibit migration kinetics and toxicological profiles different from their bulk counterparts, requiring case‐specific safety data rather than extrapolation from conventional packaging materials (Khodaei and Gholami‐Ahangaran 2023). Validation for meat‐packaging applications should additionally demonstrate that indicator performance is not compromised by direct contact with meat exudate, fat, and moisture, and that any migrating compounds remain below food‐contact safety thresholds throughout realistic shelf‐life and temperature‐abuse scenarios. Labeling clarity is equally important: without a standardized, consumer‐interpretable format for reporting indicator results, intelligent‐packaging signals risk being misread as either false reassurance or unwarranted alarm (Khodaei and Gholami‐Ahangaran 2023; Wang and Zhang 2024). Because regulatory pathways for nanomaterial‐enabled and migrating‐indicator packaging remain uneven, early and iterative engagement with regulatory authorities rather than a single pre‐market submission is likely to be necessary before such systems can be validated for commercial meat‐packaging use.

Sustainability considerations further complicate adoption. Although numerous articles advocate the use of smart packaging as the solution to food waste reduction, the incorporation of electronic or non‐biodegradable materials can lead to more harm to the environment unless the end‐of‐life is considered. This contradiction highlights the need for life‐cycle‐oriented design, where technological innovation is evaluated alongside environmental impact rather than in isolation (Wang and Zhang 2024).

### 6.6 Synthesis: Redefining Readiness for Freshness Monitoring Technologies

Combining all these difficulties, it can be pointed out that the main bottleneck in the monitoring of the freshness of meat is not the unavailability of sensing principles but the unavailability of deployment‐ready systems. Future research needs to pay less attention to the gradual increases in the limits of detection and more to robustness testing, interoperability, cost openness, and regulatory pathways.

Technologies that cannot prove to be stable in changing cold‐chain conditions, such as integrating with digital decision structures, and in line with sustainability objectives, will hardly be able to attain the results of past pilot studies. It is imperative to acknowledge these limitations to alter the field from exploratory innovation to the practical implementation.

## 7 Future Directions: From Sensing Technologies to Freshness Intelligence

The future of meat freshness monitoring will not be defined by incremental improvements in individual sensors, but by the creation of integrated and decision‐oriented systems, which will be able to work reliably even in complicated supply chains. Depending on the limitations discovered in these sections, some priority directions are developed, which should be followed in further research and industrial practice.

### 7.1 From Single Indicators to Multi‐Modal Signal Integration

A central research priority is the transition from single‐marker freshness assessment toward multi‐modal sensing frameworks. Spoilage is governed by the convergence of microbial metabolism, oxidation, and structural degradation, each producing complementary signals. Volatile sensing, optical or spectroscopic data, and environmental parameters must also be included in future systems rather than isolated indicators (Grassi and Benedetti 2023; Zhang and Yu 2023).

Sensor fusion, supported by data‐driven analytics, offers improved robustness against variability in meat type, packaging, and storage conditions. Importantly, redundancy across sensing modalities should be treated as a design requirement rather than a limitation, enabling systems to remain functional even when individual sensors degrade or drift.

Among the combinations reported to date, integrating volatile organic compound profiling with optical or colorimetric signals appears particularly promising, since VOC‐based sensing captures early microbial metabolic activity while optical signals capture the oxidative and pigment‐related changes that VOC sensing does not directly detect, together spanning a larger share of the interconnected spoilage pathways described in Section 3 (Grassi and Benedetti 2023; Wang and Zhang 2024). Adding environmental parameters such as temperature history further strengthens this combination by contextualizing sensor readings against the cold‐chain conditions that modulate spoilage rate, consistent with the shelf‐life prediction gains reported for integrated multi‐modal systems in Table 2 (Wang and Zhang 2024; Zhang and Yu 2023).

### 7.2 Predictive Freshness Modeling and Decision Support

Freshness monitoring should also develop beyond the classification by description to predictive and preventive decision support. Machine learning models should be developed not merely to label samples as “fresh” or “spoiled,” but to estimate remaining shelf life and recommend actionable interventions, such as rerouting, discounting, or withdrawal (Shtepliuk and Domènech‐Gil 2025; Wang and Zhang 2024). To achieve this, future studies must prioritize: (i) longitudinal datasets capturing real cold‐chain variability, (ii) inter‐species and inter‐packaging system validation on meat, (iii) transparent reporting of model uncertainty and failure modes. In the absence of these components, predictive models may serve to strengthen overconfidence, instead of enhancing food safety results.

### 7.3 Digital Ecosystems and Interoperability

The next generation of freshness monitoring systems will operate within digital ecosystems, where sensors, IoT platforms, and analytics engines exchange data seamlessly. Interoperability among devices, data formats, and software platforms is therefore a critical but underexplored challenge (Zhang et al. 2025). Because meat freshness‐monitoring systems currently rely on heterogeneous, vendor‐specific sensor and packaging platforms, establishing shared data schemas for freshness‐related variables (e.g., common units and reporting formats for VOC, colorimetric, and temperature‐history signals) is a prerequisite for meaningful data exchange between IoT sensing layers and enterprise decision‐support systems (Zhang et al. 2025; Zhang and Yu 2023). Edge‐computing approaches that pre‐process and standardize sensor outputs locally before transmission may help bridge heterogeneous hardware while reducing the latency and bandwidth demands associated with continuous IoT monitoring (Wang and Zhang 2024). Future research should focus on: (i) Standardized freshness data schemas, (ii) Enterprise resource planning (ERP) and HACCP system integration, (iii) Edge computing solutions that reduce data latency and cloud dependence. Such integration is essential for translating sensor outputs into operational decisions at scale.

### 7.4 Freshness‐Assessment Democratization and Different Technologies Facing the Consumer

A new and still promising trend is the creation of consumer‐facing tools of freshness assessment, especially smartphone‐based ones. Smartphones can standardize the interpretation of colorimetric indicators and have a chance to reduce subjectivity in visual inspection since they can use built‐in cameras and application‐level analytics to interpret colorimetric indicators (Ehtesabi and Afkaneh 2025).

Although analytical sensors cannot be substituted by these methods, they provide an expedient way of implementation through raising cost reduction thresholds and raising user interest. Future work should consider the issue of consumer trust, behavioral response, and alignment of sensor output and decisions to purchase, which have yet to be studied extensively.

### 7.5 Sustainability‐By‐Design and Regulatory Alignment

The freshness monitoring technologies used in the future should be developed to be sustainable and in compliance with regulations, as the main limiting factors, as opposed to being secondary. Investigations ought to take a step further by not only conducting a demonstration on the proof‐of‐concept level but also performing life‐cycle analysis, material recyclability, and end‐of‐life handling of smart packaging components (Khodaei and Gholami‐Ahangaran 2023).

Similarly, early engagement with regulatory frameworks is essential, particularly for systems incorporating nanomaterials or migrating chemical indicators. Technologies that cannot demonstrate food‐contact safety, migration control, and labeling clarity are unlikely to achieve commercial adoption regardless of technical merit (Wang and Zhang 2024).

### 7.6 Research Priorities and Field‐Level Implications

From an opinionated standpoint, the field must re‐evaluate how success is defined. Future studies should be judged not by peak accuracy under ideal conditions, but by the robustness, scalability, interpretability, and decision relevance identified as the central bottlenecks in Section 6: technical robustness and calibration stability (Section 6.1), data validity and generalizability (Section 6.2), integration into industrial workflows (Section 6.3), economic feasibility and scalability (Section 6.4), and regulatory and sustainability constraints (Section 6.5). Mass validation under realistic cold‐chain conditions and transparent cost accounting, delivered by interdisciplinary teams of food scientists, engineers, data scientists, and regulators, directly addresses these five challenges rather than adding further isolated technical advances.

Finally, the shift to freshness intelligence needs a system‐level thinking where sensing technologies are treated as one of the means to manage risks instead of being a goal in itself. The ability to integrate technological innovation and operational reality will decide how much the freshness monitoring systems of the next generation can bring significant benefits to food safety, reduction of waste, and supply‐chain resiliency.

## 8 Conclusion

Meat freshness monitoring is shifting from single‐measurement assessment toward integrated freshness intelligence. Conventional sensory, physicochemical, and microbiological methods remain essential reference tools, but their retrospective, destructive, and point‐in‐time nature limits their usefulness for real‐time, data‐driven supply‐chain management. Critically evaluating emerging technologies through a translational lens shows that analytical novelty alone does not guarantee industrial relevance: single‐indicator or peak‐laboratory‐accuracy approaches remain fragile under real cold‐chain conditions, whereas systems built on signal integration, redundancy, and contextual interpretation are better aligned with operational needs. Advancing the field will require: (i) multi‐modal sensing that captures complementary microbial, oxidative, and structural signals; (ii) predictive, decision‐oriented modeling validated externally across meat types and packaging systems; (iii) digital integration, IoT connectivity, interoperability, and edge analytics treated as a core design requirement; (iv) consumer‐facing tools, particularly smartphone‐assisted interpretation, as a practical deployment channel; and (v) early alignment with regulatory and sustainability requirements. The central contribution of this review is not a new spoilage indicator but a reframing of meat freshness monitoring as a systems‐level challenge spanning sensing, data analytics, and decision‐making, providing a critical foundation for future research and industrial implementation.

## Author Contributions

**Md Abu Rayhan:** writing – original draft, conceptualization, data curation, formal analysis, methodology. **Md. Hassan Bin Nabi:** writing – original draft, writing – review and editing, formal analysis, software, resources, investigation, visualization. **Tamanna Rahman:** writing – review and editing, formal analysis, visualization. **Md. Suhel Mia:** formal analysis, writing – review and editing. **Wahidu Zzaman:** writing – review and editing, conceptualization, supervision, project administration.

## Funding

The authors have nothing to report.

## Consent

Studies in humans and animals have not been performed.

## Conflicts of Interest

The authors declare no conflicts of interest.

## Data Availability

Data will be made available on request.
